# Prevalence and clinical characteristics of venous thromboembolism in patients with lung cancer: a systematic review and meta-analysis

**DOI:** 10.3389/fonc.2024.1405147

**Published:** 2024-08-14

**Authors:** Ying Xu, Tong Wu, Xue Ren, Jing Liu, Haibo Zhang, Defu Yang, Ying Yan, Dongyang Lv

**Affiliations:** Department of Radiation Oncology, General Hospital of Northern Theater Command, Shenyang, China

**Keywords:** lung cancer, venous thromboembolism, deep vein thrombosis, pulmonary embolism, meta-analysis

## Abstract

**Background:**

The prevalence of venous thromboembolism (VTE) is high in patients with cancer and can often present as the first symptom of malignancy. Cancer-associated VTE is one of the most important risk factors contributing to cancer mortality, making its prevention and treatment critical for patients with lung cancer.

**Methods:**

We systematically searched for observational studies that estimated the prevalence of VTE in patients with lung cancer. A comprehensive search of electronic databases, including PubMed, EMBASE and Cochrane Library, was systematically conducted from database inception through January 21, 2022. The qualities of included studies were assessed in three domains, including patient selection, comparison, and results. Random effects meta-analyses of the prevalence of VTE in lung cancer were conducted using the metaprop procedure. Chi-square test and *I* ^2^ value were used to evaluate study heterogeneity.

**Results:**

Thirty-five studies involving 742,156 patients were considered eligible for this study. The pooled prevalence of VTE among patients with lung cancer was 5% (95% CI: 0.043–0.056, *P* = 0.000). The regional prevalence of VTE was 7% (95% CI: 0.06–0.08; I^2 =^ 99.2%) in North America, 8% (95% CI: 0.06–0.10; I^2 =^ 97.6%) in Asia, 6% (95% CI: 0.04–0.09; I^2 =^ 95.9%) in Europe and 11% (95% CI: 0.07–0.15) in Australasia.

**Conclusions:**

The prevalence of lung cancer-related VTE is high and region-specific. These results of this review emphasize the importance of understanding the incidence of lung cancer-related VTE and provide argue for VTE screening of patients with lung cancer.

**Systematic Review Registration:**

https://www.crd.york.ac.uk/prospero/, identifier PROSPERO (CRD42022306400).

## Introduction

Primary lung cancer is the second most prevalent malignancy worldwide but has the highest rate of mortality (1). Although the overall survival of patients with lung cancer substantially improved in the past decade with comprehensive and updated treatment strategies, including surgery, radiotherapy, chemotherapy, targeted therapy, and immunotherapy, the risk of venous thromboembolism (VTE) in this class of patients has further increased recently (2). Venous thromboembolism can occur at any time in the course of the disease, and may at times constitute the presenting symptoms, triggering further investigations that lead to an eventual cancer diagnosis. Venous thromboembolism is the second most important risk factor contributing to mortality, after cancer itself (3). As expected, a similar trend is also observed in lung cancer (4, 5).

Venous thromboembolism can be classified into two major clinical conditions: deep vein thrombosis (DVT) and pulmonary embolism (PE). Large studies have shown that VTE is associated with worse outcomes in patients with cancer, including increased mortality. For instance, in one study, the one-year overall rate of survival in patients with lung cancer, who simultaneously had VTE, was 12%, compared to 36% in those without VTE (P < 0.001) (5). The mortality of those with VTE was 2.2-fold higher (95% confidence interval (CI) 2.05 to 2.40), while patients diagnosed with cancer within twelve months following a diagnosis of VTE had a one-year survival rate of 38%, compared to 47% percent in the control group without VTE (*P <*0.001) (6). The prevalence of cancer-related VTE varies according to patient-related factors, such as additional thrombogenic risk factors, including degree of mobility, performance status, presence of other comorbidities, past history of VTE, cancer-related factors, such as cancer location, stage, and grade, and treatment-related factors, such as whether the patient received surgery, chemotherapy, anti-angiogenic therapy, hormonal, and supportive therapy (7–9). Understanding and addressing the factors contributing to lung cancer-related VTE can significantly aid the prevention of VTE, which in turn reduces patient comorbidities and prevents reductions in performance status. This, in turn, helps to optimize the treatment schedules of cancer patients and is expected to benefit patient survival. Therefore, it is important to systematically analyze the characteristics of patients with VTE so that primary thromboprophylaxis with anticoagulant therapy and non-pharmacological interventions can be constituted appropriately.

At present, domestic and international guidelines for the prevention and management of VTE in cancer patients do not specifically outline the management of different VTE risk factors, nor do they suggest risk-factor specific or individualized management for VTE in patients suffering from different types of cancer. Therefore, assessment of the prevalence and characteristics of VTE in patients with lung cancer is of value toward optimizing the prevention and treatment of VTE in lung cancer. This may additionally aid in support of drafting updated guidelines to support these measures. The aim of this systematic review and meta-analysis is to determine the prevalence and patient-specific characteristics of VTE grouped by gender and regional characteristics of patients with lung cancer.

## Methods

This review was conducted in accordance with the PRISMA 2020 statement for systematic reviews (9). The protocol was registered in the International Prospective Register of Systematic Reviews (PROSPERO), registration number: CRD42022306400.

### Search strategy

Literature from the databases of PubMed, EMBASE, and the Cochrane Library were systematically searched without restrictions on language or geographical region, from the inception of the databases through January 21, 2022. Medical Subject Headings (MESH) terms and keywords used in the search included “venous thromboembolism” OR “VTE” OR “thromboembolism” OR “venous thrombosis” OR “pulmonary thrombosis” OR “pulmonary embolism” OR “pulmonary thromboembolism” OR “deep vein thrombosis”, AND “lung cancer” OR “pulmonary cancer” OR “lung tumor” OR “pulmonary tumor” OR “lung neoplasm*” OR “lung carcinoma” OR “pulmonary neoplasm*”. References of potential reviews were manually evaluated to identify additional studies relevant to these subjects. A full search strategy can be found in **Appendix 1**.

### Eligibility criteria

Eligible studies included observational studies reporting the prevalence of VTE among patients with lung cancer as the primary malignancy.

### Exclusion criteria

Studies excluded from this review included conference abstracts, duplicate studies, and publications with incomplete results.

### Study selection and data extraction

Two independent reviewers screened for all included studies following the primary database search. First, duplicate studies and irrelevant literature were excluded. Each reviewer independently reviewed the full texts of each potentially eligible study, and included studies based on the aforementioned inclusion and exclusion criteria. In cases of disagreement between the reviewers, discussions around the potential study eligibility were carried out with a third reviewer until consensus was drawn.

Extraction and analysis of data were carried out by the two reviewers independently using predesigned forms based on the guideline for data extraction for systematic reviews and meta-analysis (10). The following information was extracted: the names of authors, year of publication, country of study, types of observational study carried out, sample size of the studied population, study duration, patient age, VTE subclass diagnoses (PE or DVT), locations of VTE occlusions, and the number of VTE events.

### Quality assessment

The quality of the included studies was assessed using the Newcastle-Ottawa Quality Assessment Scale (NOS) (11) in three domains, including patient selection, comparison, and results. The NOS scores of observational studies are graded from 0 to 9, where higher scores indicate higher study qualities. Specifically, we grouped these scores into 3 categories: ≥7, 4–6 and 0–3, to represent studies of high, medium and low qualities, respectively.

### Statistical analysis

The metaprop procedure (12) was used to perform a meta-analysis of proportions, using Stata software (version 15.1). This approach was suitable for the analysis of binomial data and permitted the evaluation of the exact binomial and test score-based confidence intervals (CI). The meta-prop procedure provided suitable strategies for dealing with proportions close to or at the margins, where the normal approximation procedures often break down. It uses a binomial distribution to model the within-study variability and allows the Freeman-Tukey double arcsine transformation to stabilize the variances. Study heterogeneity was assessed using chi-square test and I^2^ values, *P* < 0.1 or I^2^>50% was considered to be clinically significant, and a random-effect model was adopted. Sensitivity analyses were performed to verify the robustness of the overall results and to explore sources of heterogeneity. Subgroup analyses were conducted for lung cancer-related VTE in different genders and regions. Finally, we used funnel plots and Egger’s regression test to identify publication bias.

## Results

**Figure 1:**
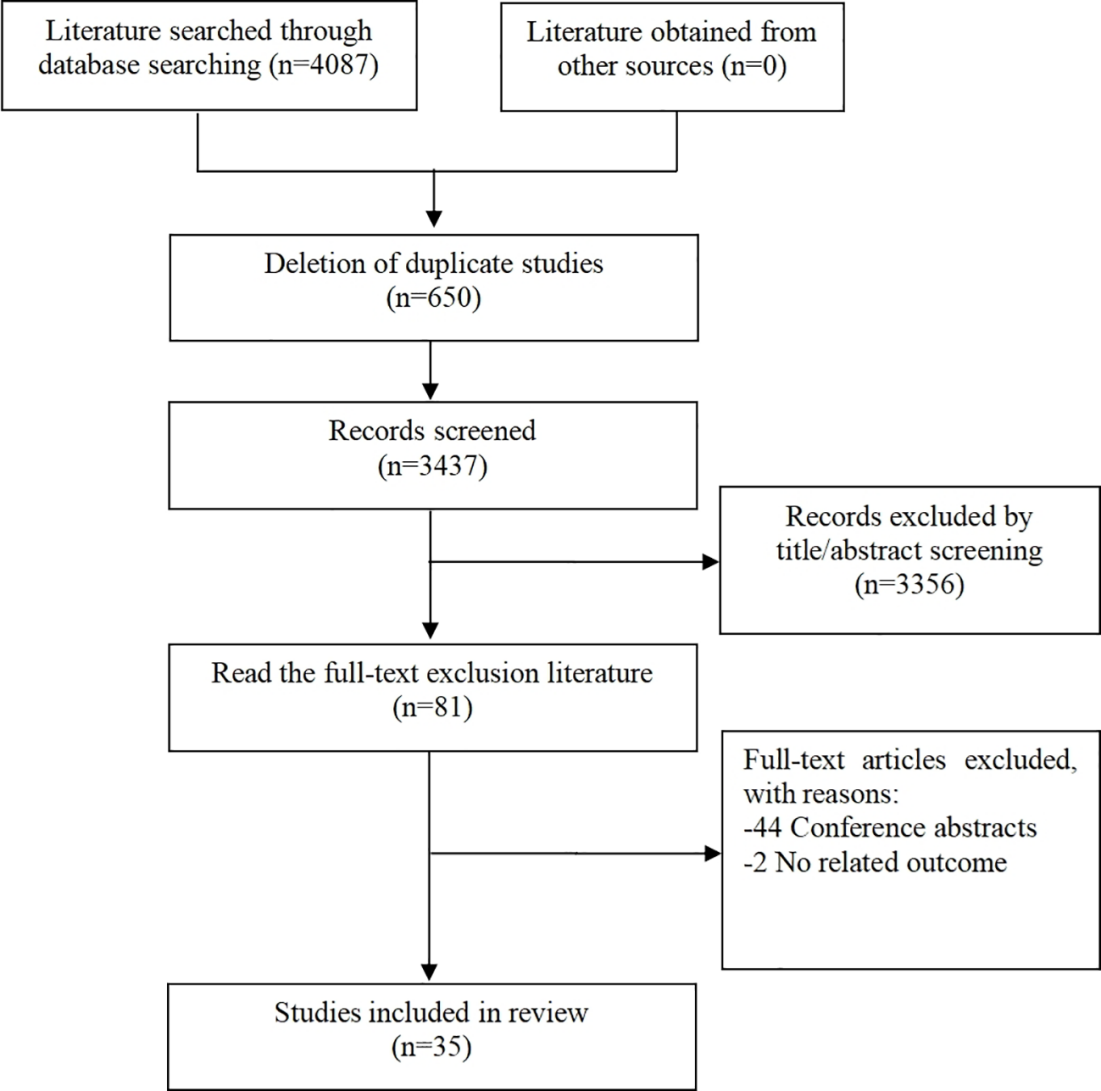
Literature screening flow chart.

Overall, a total of 4087 articles were found through the initial literature search based on the aforementioned search criteria. Of these, 650 duplicate articles and 3356 irrelevant articles were excluded after initial abstract screening. Eighty-one additional articles were excluded after full text screening. Out of the literature excluded, 44 were conference abstracts and 2 were trials with non-related outcomes. Altogether, 35 studies (13–47) were found to be eligible for this systematic review. The literature selection process is outlined in **Figure 1**.

### Study characteristics

Among the 35 studies included with VTE listed as the primary outcome (13–47), 20 were retrospective studies (13, 14, 16, 17, 19–27, 35, 37, 41, 42, 45–47), nine were cohort studies (15, 18, 28, 30–32, 38, 40, 44) and six were case-control studies (29, 32, 33, 36, 39, 43). All studies included were published from 2005 to 2021. The number of study participants varied from a minimum of 90 to a maximum of 570,304. The studies were conducted in 12 countries, including China, the United States, Canada, Japan, Germany, Australia, Czech Republic, Korea, Greece, the Netherlands, Italy, and Great Britain, which are grouped into their respective continents for ease of categorization. Specifically, there were two (16.67%) countries categorized into the North American group, three (25%) countries in Asia, six (50%) countries in Europe, and one (8.33%) country in Australasia. Thirty studies (13–19, 21–28, 30–34, 36–40, 43–47) reported the mean age of the participants, ranging from 50.34 to 72 years. International Classification of Diseases (ICD)-9 or ICD-10 codes were used to label the diagnoses of VTE in five studies (16, 18, 19, 22, 28). Most of the other 30 studies combined medical history, physical examination and imaging to aid in the diagnosis of VTE. Out of all studies included, eight studies (16, 17, 31, 36, 37, 39, 41, 42) reported on VTE, two (32, 34) reported PE only, two reported DVT only (15, 21) and 23 (13, 14, 18–20, 22–30, 33, 35, 38, 40, 43–47) simultaneously reported VTE, PE, or DVT. General characteristics of the included studies are shown in **Table 1**.

**Table 1:** Characteristics of studies included in the review.

Author	Year	Country	Study type	Sample size	Male/female	Study period	Patients	Age (years)	Diagnosis of VTE/PE/DVT	Number of VTE	Number of PE	Number of DVT	Number of PE and DVT
Mason	2005	USA	Retrospective	336	248/88	1990.1–2001.1	after pneumonectomy for malignancy	66.5 ± 7.5	history and physical examination, imaging or Doppler ultra-sonography	25	5	17	3
Tagalakis	2007	Canada	Retrospective cohort study	493	297/196	1997.1.1–2004.12.31	Non-small cell lung cancer	/	patient’s medical record with the vascular laboratory records	/	/	67	/
Chew	2008	USA	Retrospective	91933	49544/42449	1993.1–1995.12,1997.1–1999.12	primary lung cancer	50.34 ± 8.42	ICD-9-CM	3140	/	/	/
Connolly	2012	USA	Retrospective cohort study	6732	3329/3402	2005.1.1–2008.12.31	Ambulatory lung cancer	67	DVT: ICD-9 PE: ICD-9 CM	938	245	497	196
Huang	2012	USA	Retrospective database study	2242	1087/1155	2000.7.1–2008.6.30	lung cancer undergoing chemotherapy	77.4 ± 10.8	DVT: ICD-9-CM PE: ICD-9-CM	235	36	168	31
Yang	2012	China	Retrospective	1001	656/345	2009.1–2009.12	Postoperative in Lung Cancer	73.0 ± 10.6	Spiral computer tomography, pulmonary angiography, and color Doppler ultrasound examination	53/1001	8	41	4
Crolow C	2013	Germany	Retrospective	1940	1209/731	2008.1–2010.12	lung cancer	/	ICD-10-GM	148	/	82	/
Alexander	2014	Australia	Retrospective	222	131/91	2011.1.7–2012.6.30	lung cancer	/	Radiologically confirmed symptomatic or clinically unsuspected	24	16	4	1
Bohdan	2014	Czech Republic	Retrospective	950	600/350	2006.1–2010.5	primary lung cancer	64.80 ± 10.67	Standard diagnostic procedures	91	34	58	13
Kourelis	2014	USA	Retrospectively	727	376/351	1998.1–2011.12	lung cancer	67.9	Venogram, computed tomographic scan or magnetic resonance imaging scans	95	34	35	6
Lee	2014	Korea	Retrospectively	1998	1338/660	2006.1–2010.6	non-small cell lung cancer	66.7	Computerized tomography, CT angiography, Doppler ultrasonography, and conventional angiography or nuclear medicine (ventilation/perfusion scans)	131	72	16	24
Zhang	2014	China	Retrospective	673	486/187	2009.1–2011.1	lung cancer	72	Venous ultrasound imaging or a CT venous angiogram	89	33	42	14
Steuer	2015	USA	Retrospective cohort study	570304	274543/248187	2006–2010	hospitalized lung cancer	61	ICD-9	20672	14030	/	/
Wang	2015	China	Case-control study	4726	/	2004.1–2013.7	non-small cell lung cancer	65	Duplex ultrasound or computed tomography angiography	61	5	45	11
Walker	2016	UK	Cohort study	10598	/	1997.4.1–2006.12.31	lung cancer	60	Relevant medical code in either the CPRD and HES	364	/	/	/
Ma	2017	China	Retrospective case-control study	90	60/30	2010.1–2015.1	lung cancer	68	PE:echocardiography, computed tomography, magnetic resonance imaging, or ventilation/perfusion	/	30	/	/
Shen	2017	China	Case- control study	1560	/	2012.3–2015.5	non-small cell lung cancer	66.7	DVT: venous ultrasound image or a CT venous angiogram. PE: CT pulmonary angiogram or a ventilation-perfusion scan	32	12	14	6
Li	2018	China	Rretrospective cohort study	11474	/	2012.1–2015.7	Lung cancer surgery	58	PE:computed tomographic pulmonary angiograph, or echocardiography	/	56	/	5
Thomas	2018	USA	Retrospective	14308	/	2005–2015	After lung cancer resection	66.5 ± 7.5	Computer tomography exam, transesophageal echocardiogram, pulmonary arteriogram, CT angiogram, or any other definitive imaging modality	234	116	150	32
Fu	2019	China	Case-control study	200	95/105	/	Lung cancer after chemotherapy	/	Doppler ultrasonic examination	40	/	/	/
Makoto	2019	Japan	Retrospective	682	449/233	2014.1–2016.12	lung cancer	50.34 ± 8.42	DVT: venous ultrasonography or contrast enhanced computed tomography. PE: CT pulmonary angiography	71	/	/	/
Liu	2020	China	Propensity cohort	106	57/49	2006.1–2016.12	Elderly with lung cancer	67	CT pulmonary angiography features.	/	53	31	/
Suzuki	2020	Japan	Retrospective cohort	1471	1028/443	2008–2017	lung cancer	77.4 ± 10.8	Contrast-enhanced computed tomography or echography of the lower limb veins, or mismatch of lung ventilation/perfusion scintigraphy	28	5	14	9
Wang	2020	China	Retrospective	600	339/261	2018.1–2019.8	lung cancer after surgery	73.0 ± 10.6	Computer tomography pulmonary angiogram.	77	/	/	/
Akhtar-Danesh	2021	Canada	Retrospective	12626	5923/6703	2007–2017	Lung cancer surgery	/	VTEs were investigated and subsequently diagnosed based on symptoms	345	/	/	/
Cui	2021	China	Case-control study	100	43/57	2013.1–2019.5	lung cancer	/	Pulmonary Computed Tomography Angiography	/	40	9	/
Dimakakos	2021	Greece	Retrospective	217	176/41	2015.1–2018.6	Small cell lung cancer	64.80 ± 10.67	Computed tomography pulmonary angiograph, ventilation/perfusion lung scintigraphy, or venous duplex ultrasonography	9	6	3	/
Deschênes-Simard	2021	Canada	Retrospective multicentric cohort study	593	322/271	2013.6–2020.9	Non-small cell lung cancer	67.9	Doppler ultrasonography, computed tomography pulmonary angiograph, and magnetic resonance imaging	59	36	16	/
Takemoto	2021	Japan	Retrospective	944	563/381	2013.6–2018.12	Lung cancer before surgery	66.7	Venous ultrasonography and computed tomography	/	1	91	/
Zhang	2021	China	Retrospective	952	418/534	2010.5–2018.8	adenocarcinoma of the lung undergoing surgical resection	72	DVT: venous ultrasound or computed tomography venous angiogram, PE: computer tomography or ventilation-perfusion scanning	100	7	86	7
Blom	2004	Netherlands	Retrospective	537	429/108	1990.1–2000.12	lung cancer	61	The medical records and consult the general practitioner of the patient	39	15	17	7
Patel	2009	Canada	Retrospective	186	122/64	1996.1–2007.12	multimodality therapy for lung cancer	65	PE: computed tomography scan or V/Q scan, DVT: Doppler ultrasonography	23	/	/	/
Buosi	2013	Italy	Retrospective	307	245/62	2008.1–2011.5	Non-small cell lung cancer	60	Symptomatic DVT, echocolordoppler or computed tomography; symptomatic PE, CT or pulmonary angiography; asymptomatic DVT, echocolordoppler or CT; asymptomatic PE, CT or pulmonary angiography	/	/	36	/
Agzarian	2015	Canada	Prospective cohort study.	157	72/85	2013.6–2014.12	After lung cancer resection and discharge	68	Radiographic screening	19	14	3	/
Li	2020	China	Case-control study	171	75/96	2016.7–2017.12	After radical operation of non-small cell lung cancer	66.7	Doppler ultrasonography and computer tomography pulmonary angiography	23	/	/	/

### Quality bias assessment

The qualities of all eligible studies in our meta-analysis, evaluated using the NOS scale, are outlined in **Table 2**. Twenty-eight (80%) studies (13–16, 19–26, 29–31, 33, 34, 36–39, 41–47) scored seven or higher, which were classified as high quality. Seven (20%) studies (17, 18, 27, 28, 32, 35, 40) scored between four and six inclusive and were classified as medium quality. There were no low-quality studies scoring three or below. Our 35 studies had a mean NOS score of 7.486, indicating high quality of the studies included in this series of studies reviewed.

**Table 2:** Risk of bias for included studies.

COHORT STUDIES					
First author	Year	Selection	Comparability	Outcome	Overall quality score
Mason	2005	★★★★	★★	★★	8
Tagalakis	2007	★★★★	★★	★	7
Chew	2008	★★★	★	★★★	7
Connolly	2012	★★★	★	★★	6
Huang	2012	★★★★	★★	★★	8
Yang	2012	★★★	★★	★★	7
Crolow	2013	★★★★	★★	★★	8
Alexander	2014	★★★	★★	★★★	8
Bohdan	2014	★★★	★	★★★	7
Kourelis	2014	★★★	★★	★★	7
Lee	2014	★★★★	★	★★	7
Zhang	2014	★★★	★	★★	6
Steuer	2015	★★★	★	★★	6
Walker	2016	★★★★	★★	★★	8
Li	2018	★★★	★★	★★★	8
Thomas	2018	★★★	★	★★	6
Makoto	2019	★★★	★★	★★	7
Liu	2020	★★★★	★★	★★	8
Suzuki	2020	★★★	★	★★	6
Wang	2020	★★★★	★★	★★★	9
Akhtar-Danesh	2021	★★★★	★★	★★	8
Dimakakos	2021	★★★	★★	★★★	8
Deschênes-Simard	2021	★★★★	★★	★★★	9
Takemoto	2021	★★★★	★★	★★★	9
Zhang	2021	★★★★	★★	★★	8
Blom	2004	★★★	★★	★	7
Patel	2009	★★★	★	★★	6
Buosi	2013	★★★★	★★	★★	8
Agzarian	2015	★★★★	★★	★★★	9
CASE CONTROL STUDIES					
First author	Year	Selection	Comparability	Exposure	Overall quality score
Wang	2015	★★★	★★	★★	7
Ma	2017	★★★	★	★★	6
Shen	2017	★★★★	★★	★★	8
Fu	2019	★★★	★★	★★★	8
Cui	2021	★★★★	★★	★★★	9
Li	2020	★★★★	★★	★★	8

The rating criteria are as follows: ★ represents 1 score, ★★ represents 2 scores, ★★★ represents 3 scores, and ★★★★ represents 4 scores.

### Prevalence of VTE in patients with lung cancer

**Figure 2:**
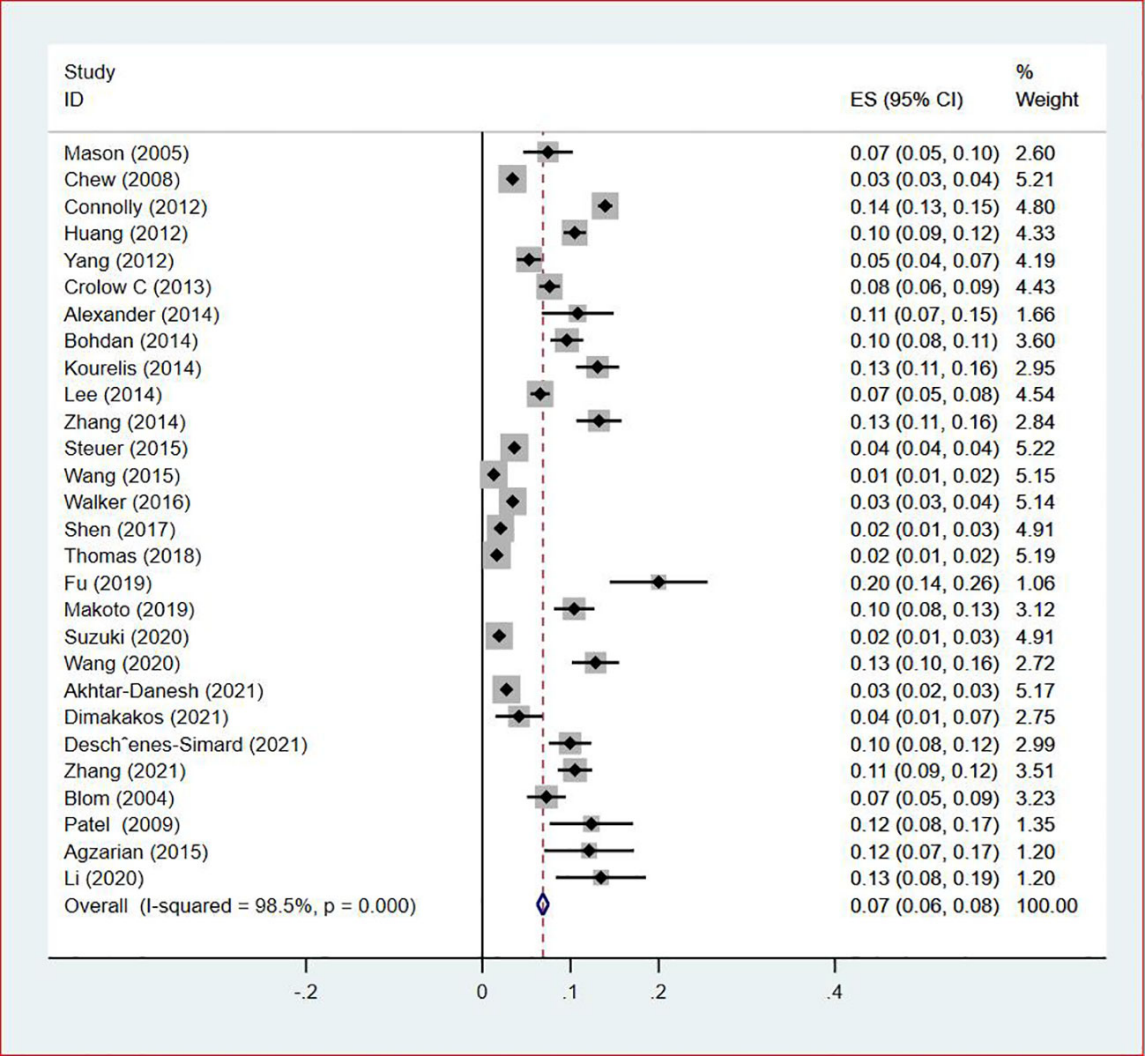
Forest plot showing the prevalence of VTE in patients with lung cancer.

**Figure 3:**
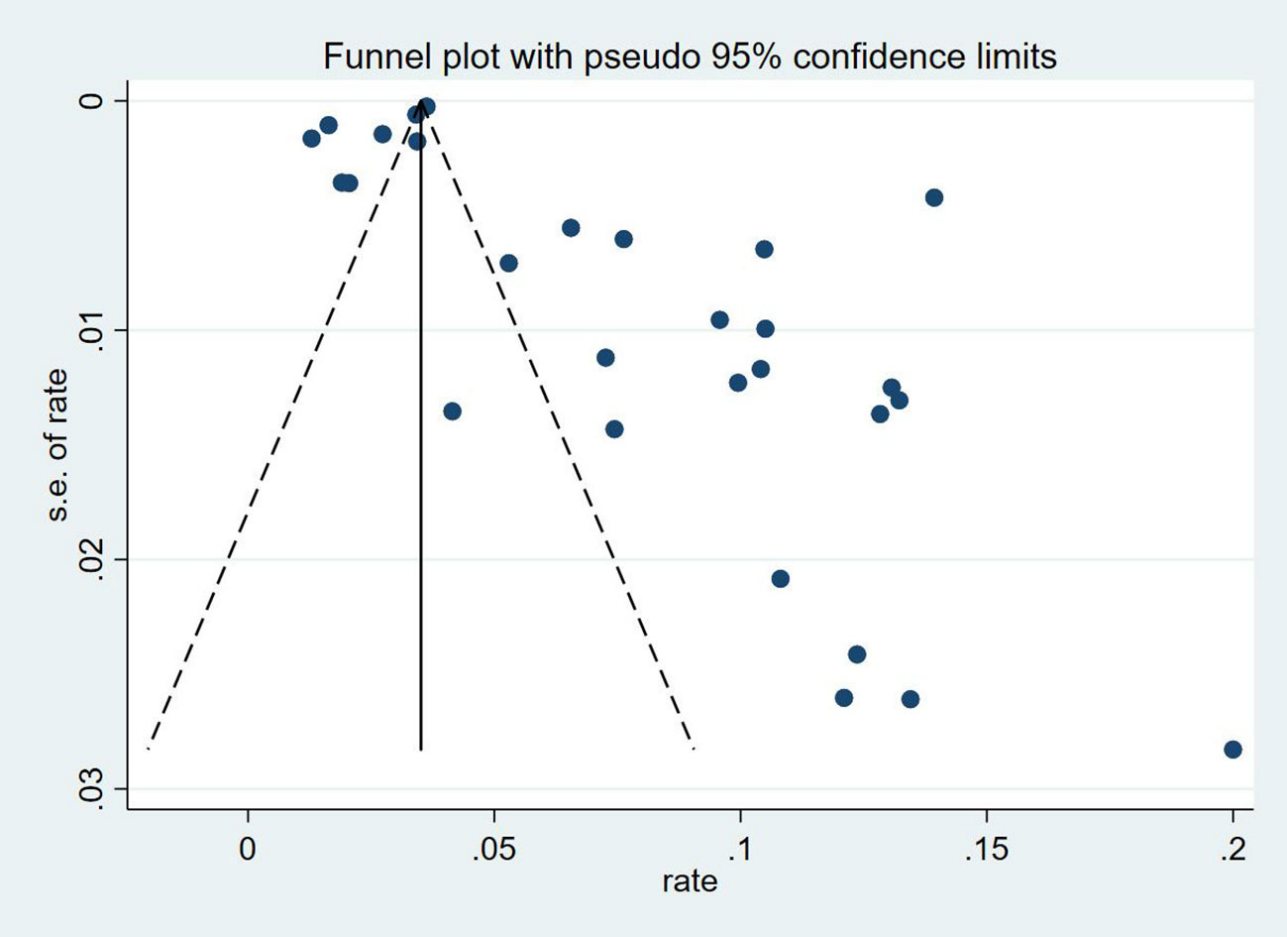
Funnel figure demonstrating the prevalence of VTE in patients with lung cancer.

**Figure 4:**
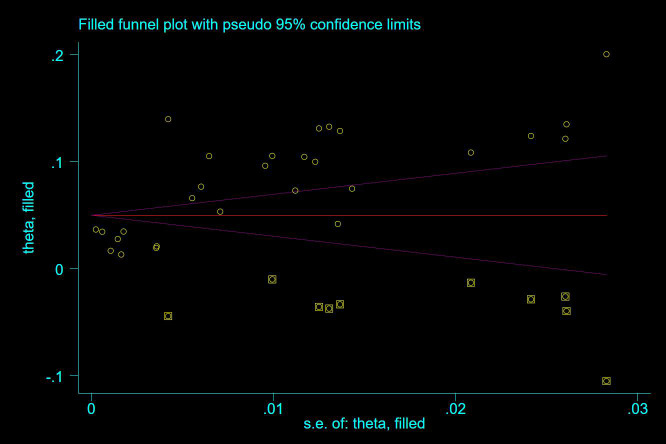
Clipped funnel plot demonstrating the prevalence of VTE in patients with lung cancer.

Thirty-five studies (13–47), 29 (82.86%) (13, 14, 16–31, 33, 35–37, 39–42, 44, 45, 47) reported the prevalence of VTE among patients with lung cancer, ranging from 1% to 20%, with an estimated pooled prevalence of 7% (95% CI: 0.06–0.08; I^2^ = 98.5%), as shown in **Figure 2**. Owing to the significant heterogeneity between different study designs (I^2^ = 98.5%, *P* = 0.0002), a sensitivity analysis was performed by omitting each study individually to explore the sources of heterogeneity. The effect of lung cancer on the overall prevalence of VTE was relatively robust after removing three studies (16, 18, 28) with high risks of bias (**Appendix 2**). A funnel plot on this revealed significant publication bias (**Figure 3**). Publication bias was found to be high in this meta-analysis, as demonstrated by Egger’s regression tests (*P*=0.034). Using the clipping method, we found the adjusted prevalence of VTE in patients with lung cancer to be 5% (95% CI: 0.043–0.056, *P* < 0.001). This was not significantly different from the pre-adjusted prevalence of 7% (95% CI: 0.062–0.075, < 0.001), indicating the prevalence of VTE was not significantly affected by publication bias (**Figure 4**).

### Sub-group analysis of VTE in patients with lung cancer

**Figure 5:**
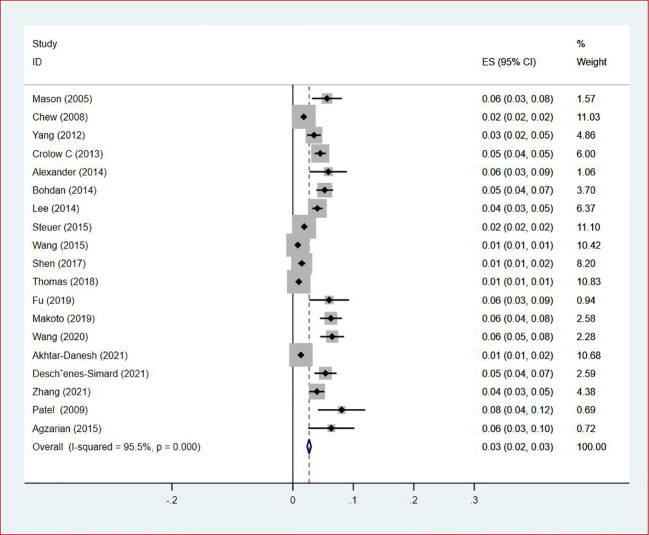
Forest plot demonstrating the VTE prevalence in male patients with lung cancer.

**Figure 6:**
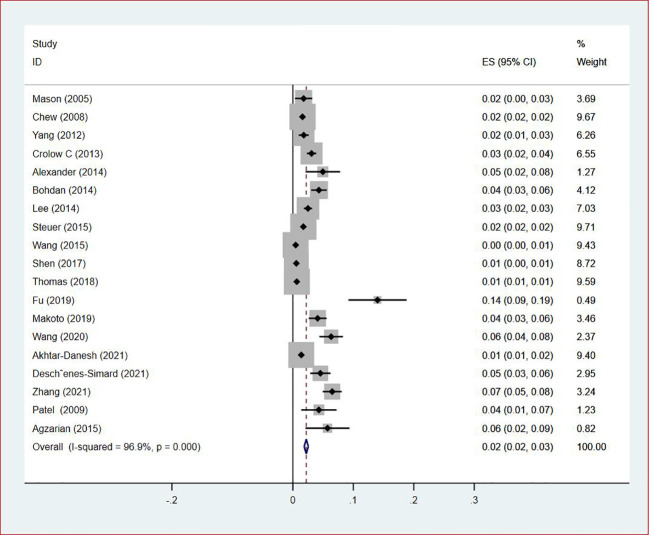
Forest plot demonstrating the VTE prevalence in female patients with lung cancer.

**Figure 7:**
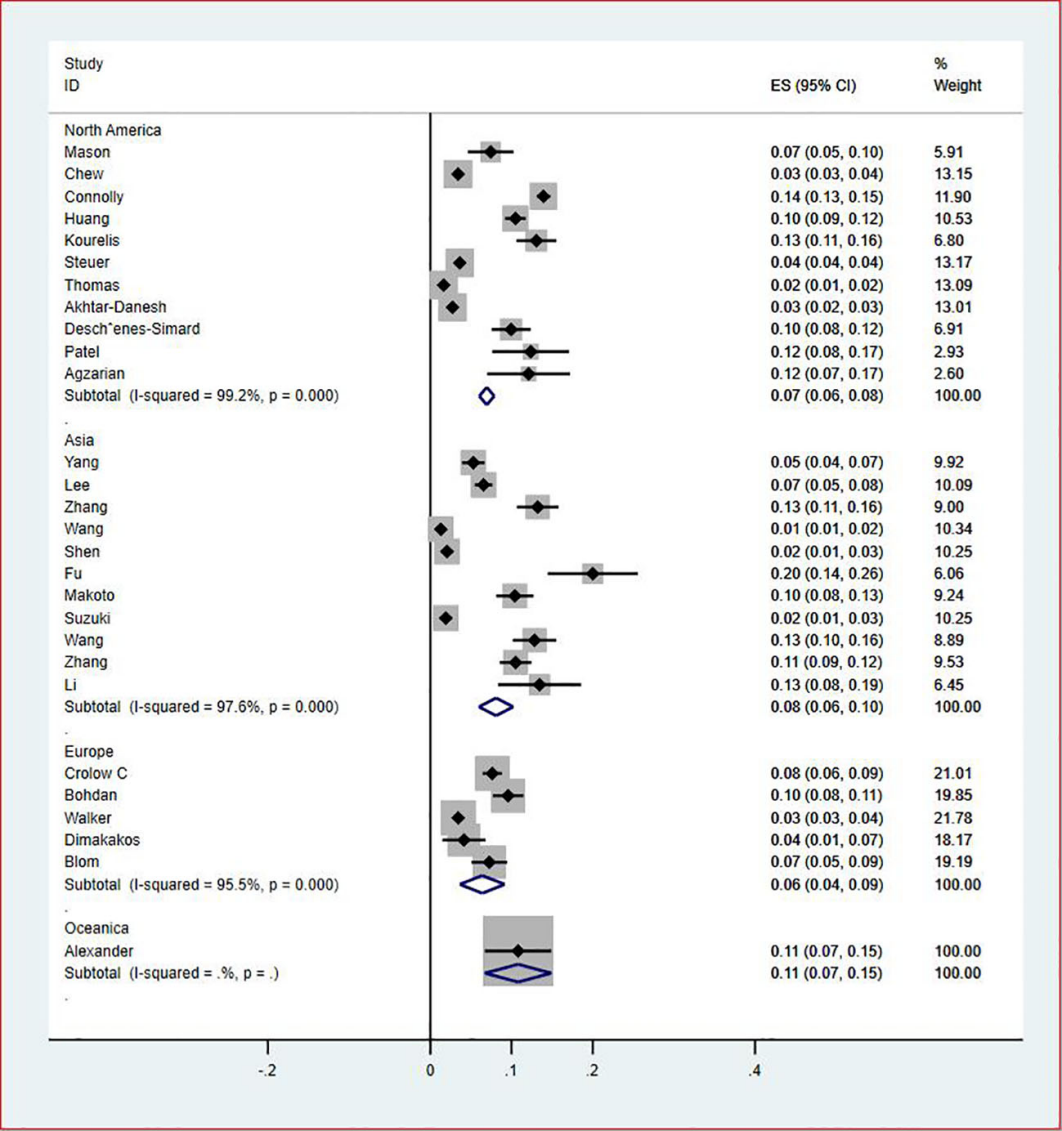
Forest plot demonstrating regional prevalence of VTE in patients with lung cancer.

Of all included studies, 19 studies (14, 16, 17, 20, 22–24, 26, 28–30, 33, 35–37, 41, 42, 44, 47) reported the prevalence of VTE by gender. The pooled prevalences of VTE in male and female patients were 3% (95% CI: 0.02–0.03; I^2^ = 95.5%) and 2% (95% CI: 0.02–0.03; I^2^ = 96.9%), respectively (**Figures 5**, **6**). Eleven studies were performed in North America and Asia each, five were conducted in Europe and one in Australasia. The regional prevalence of VTE in patients with lung cancer was 7% (95% CI: 0.06–0.08; I^2^ = 99.2%) in North America (15–19, 25, 28, 30, 35, 42, 44), 8% (95% CI: 0.06–0.10; I^2 ^= 97.6%) in Asia (20, 26, 27, 29, 33, 36, 37, 39–41, 47), 6% (95% CI: 0.04–0.09; I^2 ^= 95.9%) in Europe (13, 22, 24, 31, 45) and 11% (95% CI: 0.07–0.15) in Australasia (23), respectively (**Figure 7**).

### Prevalence of DVT in patients with lung cancer

**Figure 8:**
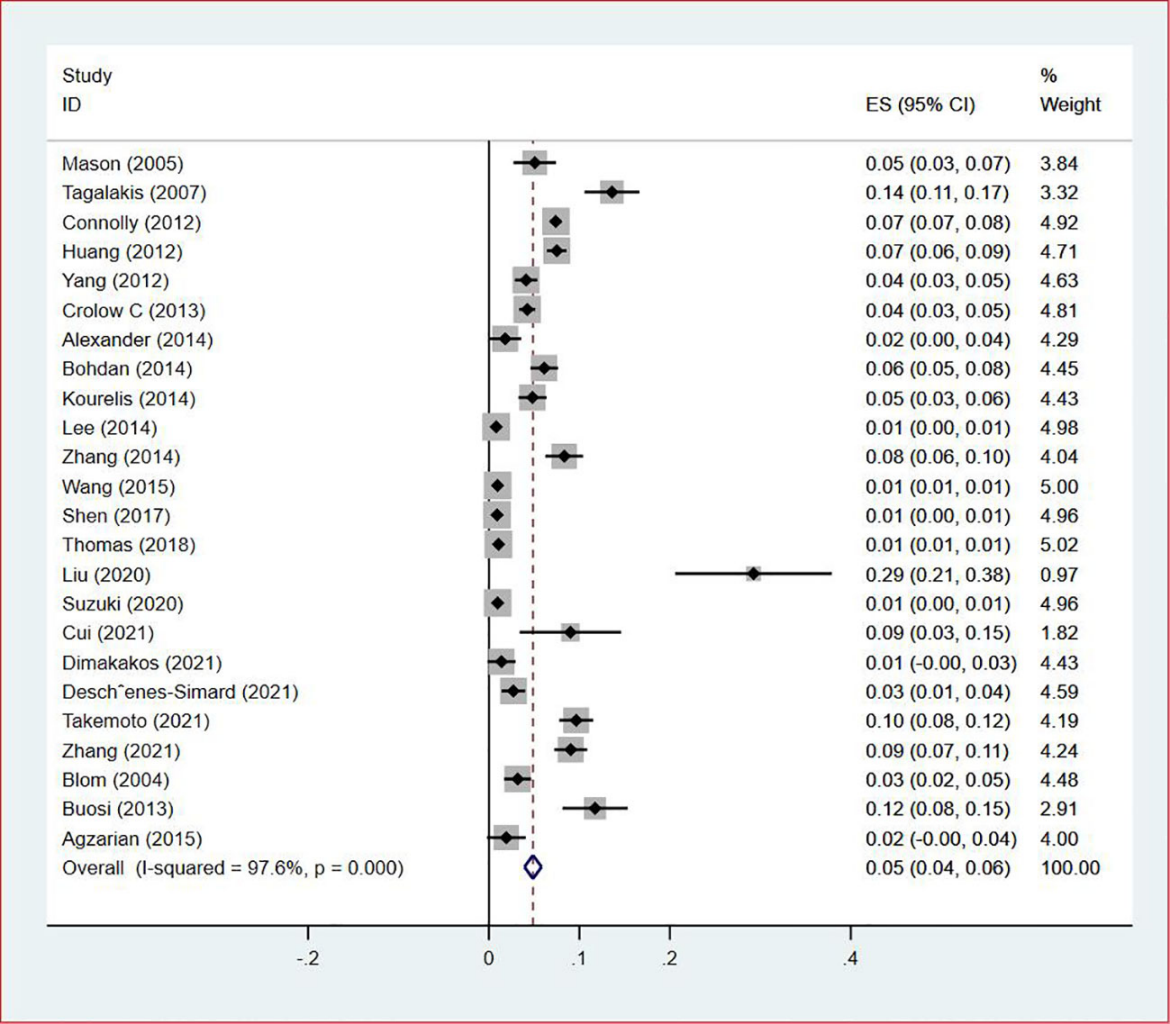
Forest plot demonstrating DVT prevalence in lung cancer.

**Figure 9:**
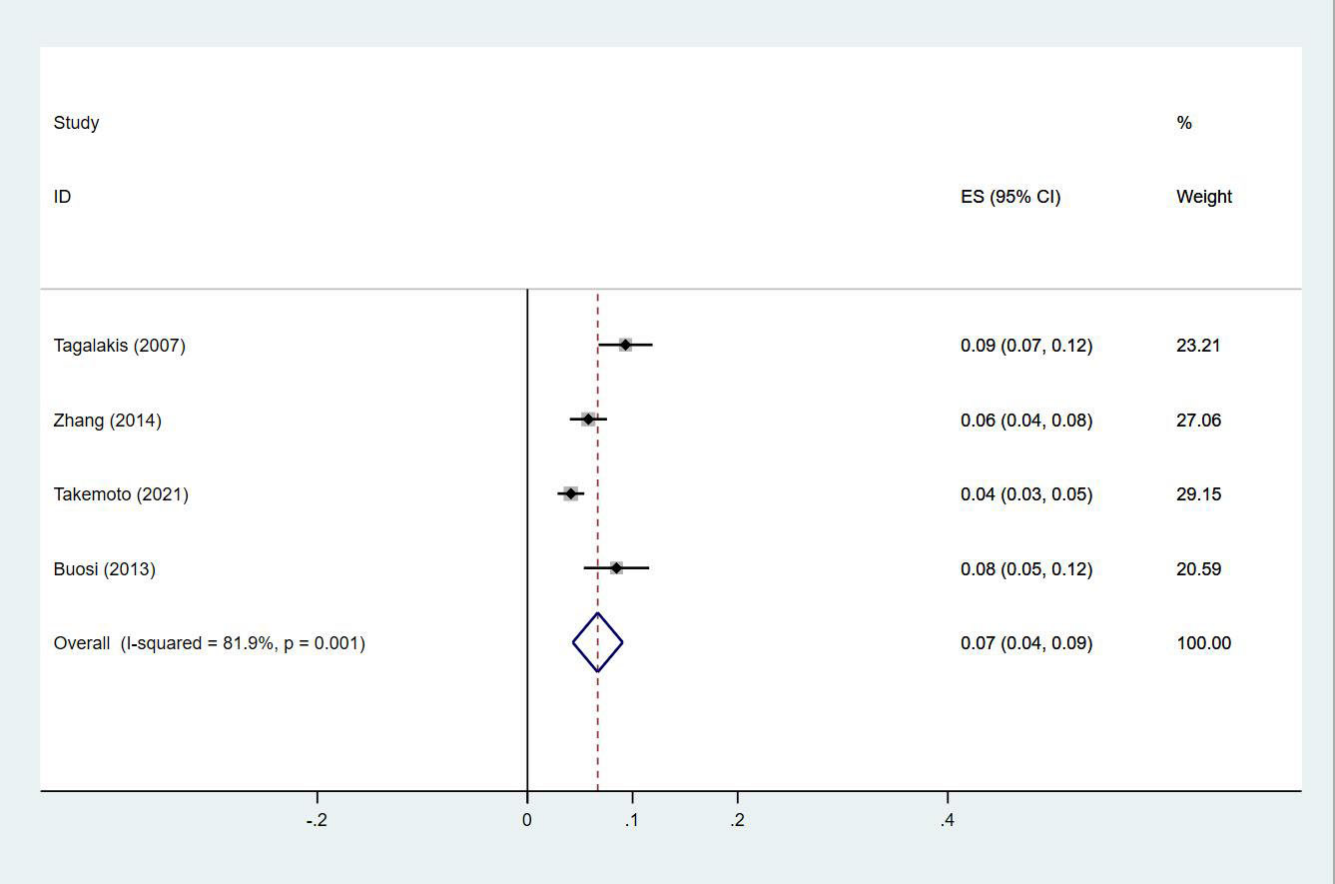
Forest plot demonstrating DVT prevalence in male patients with lung cancer.

**Figure 10:**
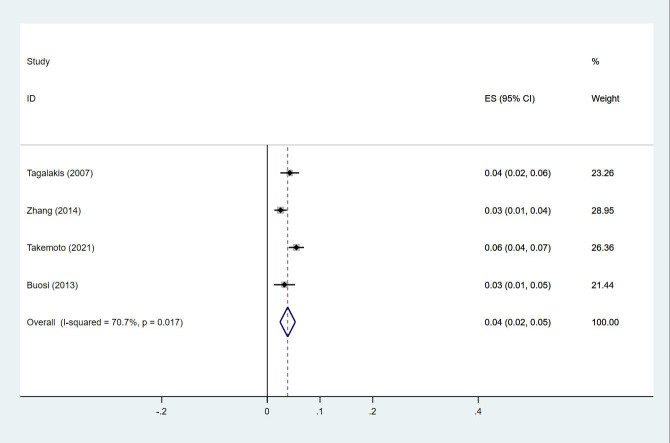
Forest plot demonstrating DVT prevalence in female patients with lung cancer.

Twenty-four studies (13–15, 18–27, 29, 30, 33, 35, 38, 40, 43–47) reported the prevalence of DVT in patients with primary lung cancer, which ranged from 1% to 29%. The pooled prevalence was 5% (95% CI: 0.04–0.06; I^2 ^= 97.6%) (**Figure 8**). Four studies (15, 21, 27, 46) simultaneously reported the proportions of DVT grouped by gender, with the pooled prevalences in men and women being 7% (95% CI: 0.04–0.09; I^2 ^= 81.9%) and 4% (95% CI: 0.02–0.05; I^2 ^= 70.7%), respectively (**Figures 9**, **10**).

### Prevalence of PE in patients with lung cancer

**Figure 11:**
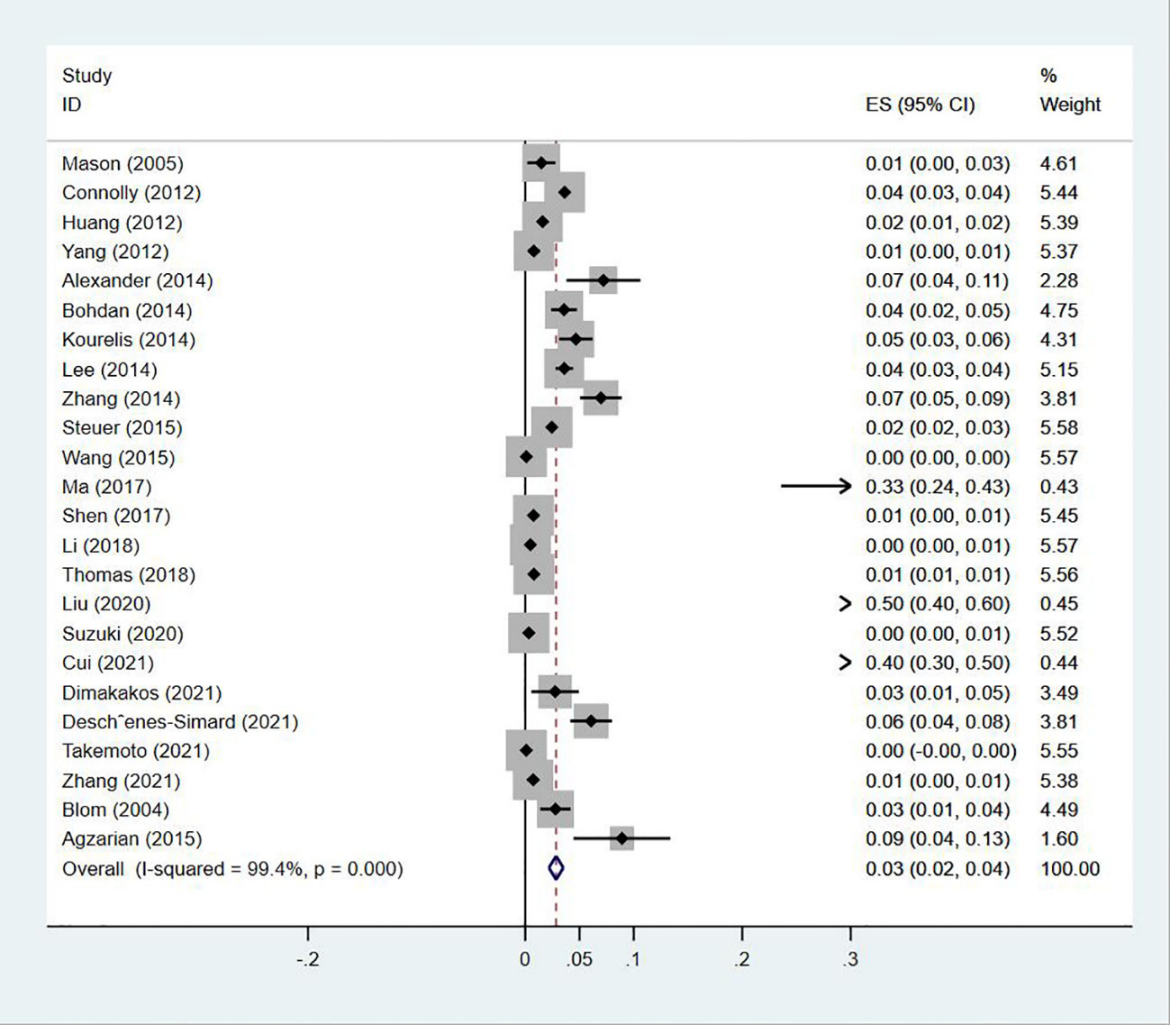
Forest plot demonstrating PE prevalence in lung cancer.

**Figure 12:**
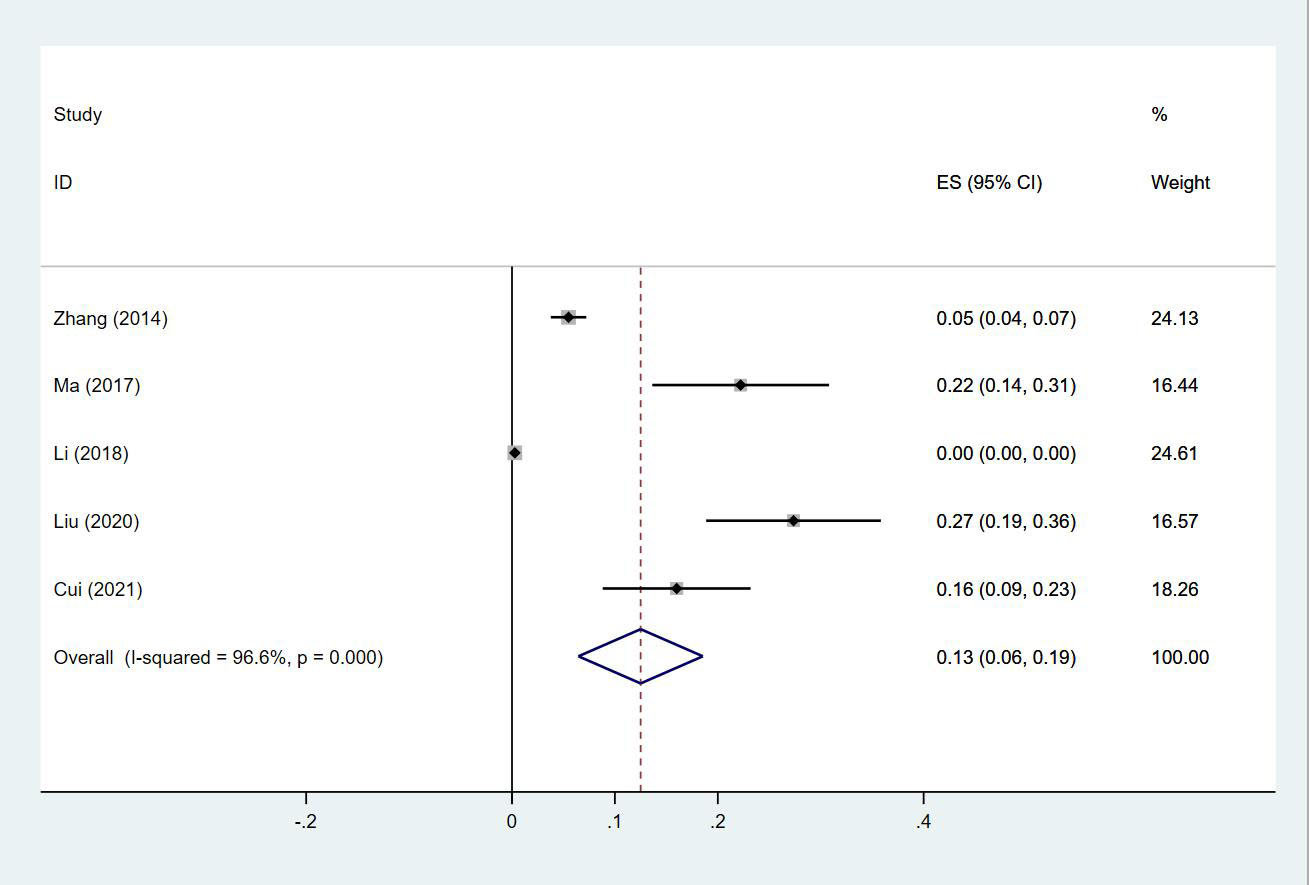
Forest plot demonstrating PE prevalence in male patients with lung cancer.

**Figure 13:**
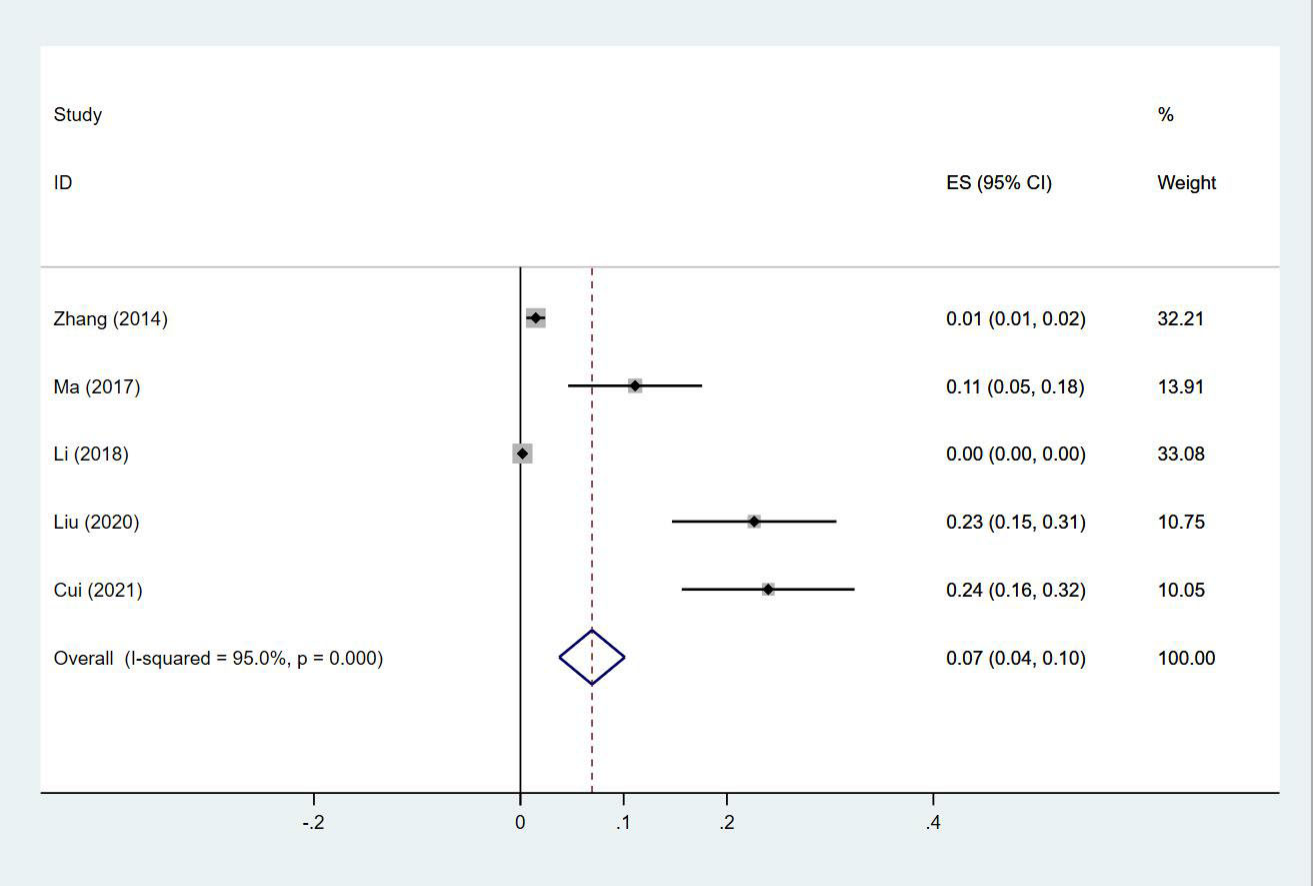
Forest plot demonstrating PE prevalence in female patients with lung cancer.

Twenty-four (13, 14, 18–20, 23–30, 32–35, 38, 40, 43–47) studies reported the prevalence of PE in patients with lung cancer, which ranged from 0% to 50%. The pooled prevalence was 3% (95% CI: 0.02–0.04; I^2 ^= 99.4%) (**Figure 11**). Five studies (27, 32, 34, 38, 43) reported the prevalence of VTE in men and women, with the pooled prevalences being 13% (95% CI: 0.06–0.19; I^2 ^= 96.6%) and 7% (95% CI: 0.04–0.10; I^2 ^= 95.0%) in men and women, respectively (**Figures 12**, **13**).

### Prevalence of DVT and PE in patients with lung cancer

**Figure 14:**
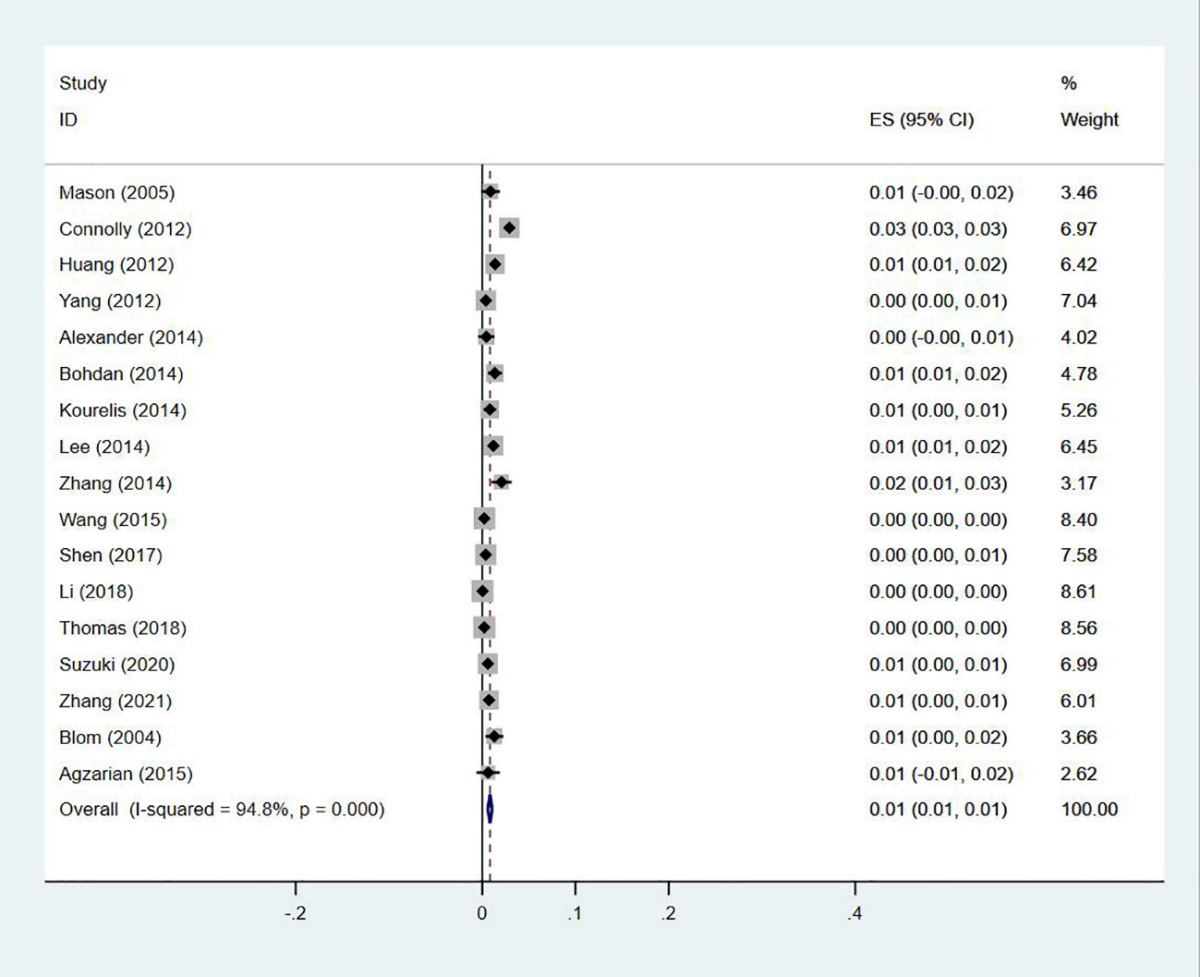
Forest plot demonstrating DVT and PE prevalence in lung cancer.

Seventeen studies (13, 14, 18–20, 23–27, 29, 30, 33–35, 40, 47) reported the prevalence of the simultaneous occurrence of DVT and PE in lung cancer, ranging from 0% to 3%, with a pooled prevalence of 1% (95% CI: 0.01–0.01; I^2 ^= 94.8%) (**Figure 14**).

### Sites of occlusion of VTE in acute exacerbations of chronic obstructive pulmonary disease (AECOPD)

Nine studies (14, 15, 23, 25, 27, 30, 41, 46, 47) reported on the specific sites of occlusion for VTE events, in which two (13, 33) reported that of both PE and DVT, whereas others (14, 15, 23, 25, 40, 41, 46) focused on either PE, DVT or VTE. Details on the sites of occlusion for the various VTE events are shown in **Table 3**.

**Table 3:** Location characteristics of VTE included in the study.

Author	Year	VTE	Cases	Location
Mason	2005	DVT	17	14 upper extremity, 15 lower extremity, and 4 central (iliac and vena cava) cases.
Tagalakis	2007	DVT	67	lower extremity, with 32 episodes limited to the distal leg veins and 35 involving the proximal leg veins
Alexander	2014	PE	16	8 proximal and 5 segmental
Lee	2014	VTE	131	Inferior or superior vena cava, neck vein, portal vein and other sites
Zhang	2014	DVT	56	distal leg vein, proximal leg vein
		PE	47	segmental or subsegmental pulmonary artery, and above the segmental pulmonary artery
Suzuki	2020	DVT	14	the superior vena cava, jugular, subclavian, or great saphenous vein (10 had central type thrombosis, 6 had peripheral type lower limb vein thrombosis, 1 had great saphenous vein thrombosis (with concomitant PTE), 3 had superior vena cava thrombosis, 2 had subclavian vein thrombosis, and 1 had jugular vein thrombosis)
Wang	2020	VTE	77	73 patients with lower extremity muscular calf vein thrombosis and 4 patients with proximal DVT
Takemoto	2021	DVT	91	17 patients had proximal DVT (proximal alone, 10; proximal and distal, 7) and 74 patients (81.3%) had distal DVT alone. Regarding laterality, 46 patients had only left-sided DVT, 27 had only right-sided DVT, and 18 had bilateral DVT
Zhang	2021	DVT	86	Lower extremity and pelvis 85, and upper extremity and neck 1
		PE	7	Segmental/subsegmental 6, and above segmental 1

## Discussion

This systematic review included 35 studies with 742,156 individuals. It demonstrated that the number of patients with VTE, as previously reported, is substantial in those suffering from lung cancer, with gender and regional variances. In evaluating the prevalence and characteristics of all VTE, DVT, and PE, we found the prevalence of VTE was the highest in Australasia and lowest in Europe. The prevalence of VTE was higher in male patients with lung cancer. DVT predominantly occurred in the distal lower extremities, while PE mostly occurred in segmental or subsegmental pulmonary arteries. This systematic review summarized the prevalence of VTE in patients with lung cancer, which provides a theoretical basis in constructing guidelines for the screening, prevention and management of thromboembolism in lung cancer.

VTE is one of the most important comorbidities leading to increased morbidity and mortality in cancer patients (7). Approximately 15% of patients are diagnosed with at least one VTE event in the course of their cancer (48). Primary lung cancer remains the leading cause of cancer death worldwide, which is further compounded by the high prevalence of VTE that both worsens the performance status of affected patients and complicates their cancer management. Understanding the characteristics of lung cancer-associated VTE is therefore of the utmost importance.

The association of race with VTE may reflect the different regional rates of VTE. It is widely understood that patients of Asian ethnicity have lower risks of VTE, where several ethnic comparative studies reported lower rates of thromboembolism affecting the venous system in patients of Asian descent in the United States when compared to Caucasians and African Americans (49). A study found at most a modest association of race with risk of VTE, particularly once comorbid conditions and socioeconomic status were accounted for in three large US cohort studies including 51,149 individuals and 916 VTE events. VTE rates were higher among black persons than white persons in the United States (risk ratio, 1.81; 95% confidence interval, 1.20–2.73) and significantly higher among black persons in the Southeast than black persons in the rest of the United States (risk ratio, 1.63; 95% confidence interval, 1.08–2.48), whereas they were not higher among white persons (risk ratio, 0.83; 95% confidence interval, 0.61–1.14) (50). A recent investigation found that the incidence of VTE varied across ethnic groups, with the highest incidence identified among African Americans (4.02%), followed by non-Hispanic Caucasians (2.98%), Hispanics (2.08%) and Chinese (0.79%) participants (51). The association between BMI and venous thromboembolism was strongest among non-White women with the higher incidence for obese compared with non-obese patients. Risk of venous thromboembolism increased with age for all race/ethnicities. Most of the above studies of geographic or racial incidence of VTE have used the Atherosclerosis Study Cohort, the Cardiovascular Health Study Cohort, and the Causes of Geographic and Racial Differences in Stroke Study Cohort, among others. Fewer lung cancer study cohorts are currently available. We used the existing literature to summarize the geographic and racial incidence of VTE associated with lung cancer. Our meta-analysis found that the overall prevalence of VTE in patients with lung was 5%, with a male predominance. The incidence of VTE varied significantly across regions and appeared to be the highest in Australasia, though only one study from Australia was included. Patients in North America and Asia had similar incidences of VTE, while Europeans had the lowest incidence of VTE. Interestingly, previous studies have not shown these meaningful conclusions. Reasons for such potential differences between different ethnicities are unclear. A few potential confounding factors may be the varying risk factors occurring in patients of different ethnicities, with obesity, diabetes mellitus, and elevated factor VIII being more common in African Americans. Similarly, genetic polymorphisms, such as factor V Leiden and mutations in the prothrombin gene 20210A are more common in Caucasians (52), which may explain the high incidence of VTE in Australia, with Caucasians being the largest ethnicity in this study. These findings may promote widespread adoption of risk assessment tools, and target prophylactic anticoagulation. Regional differences suggest the need to adapt prevention strategies to local epidemiological data and to increase medical resources, such as specialized training, equipment, and anticoagulants, in high-risk areas. In addition, raising patients’ awareness of VTE risk can help improve adherence to preventive measures.

DVT and PE are the most common types of VTE, with PE closely associated and often occurring secondary to DVT. In our study, 17 studies reported a low combined prevalence of 1% of DVT and PE, which was consistent with previous reports. DVT mostly occurred in the distal lower extremities, while PE mostly occurred in segmental or subsegmental pulmonary arteries. In one of our studies (41), 28 of 1471 patients with lung cancer were diagnosed with VTE, including five patients with PE alone (0.34%), nine with PE diagnosed following DVT (0.61%), and 14 patients with DVT alone (0.95%). The incidence of PE occurring following diagnoses of DVT was 44.7%, and patients with risk factors accounted for 88.3%. The biggest risk factors for VTE were prolonged bed rest, surgery, hyperlipidemia, hypertension, malignancy, increased hemoglobin, heart disease, trauma, and femoral vein puncture. The number of risk factors increases with age and patient comorbidities. The most common clinical manifestations of DVT were lower limb swelling (91.3%) and pain (58.3%). The femoral vein is the most commonly involved site of occlusion for DVT in lung cancer, which also has the highest association with secondary PE. Therefore, for this group of high-risk patients, we need to closely monitor these risk factors, implement stratified management, and take appropriate preventive and therapeutic measures. This will help reduce the risk of DVT and PE, which are of critical clinical importance.

The incidence of DVT in cancer ranges from 1% to 11%, and is commonly indicative of poor prognosis (53). Among cancer patients presenting with DVT, cancers of the lung, prostate, breast, and colorectum are the most common, which comprise approximately 10% to 15% of all patients diagnosed with DVT (54). In our study, the prevalence of DVT was 5%, with a prevalence of 7% and 4% in men and women, respectively. The prevalence of DVT is higher in men, with male gender being an independent risk factor for DVT (17). As demonstrated in another study, the cumulative incidence of PE was 2.2% among 8014 patients with lung cancer (55). PE is associated with increased mortality in lung cancer (5). In a large registry study, 3% of deaths in cancer patients were PE-related, versus 1% of deaths in patients not suffering from cancers (56). In our study, the prevalence of PE was 3%, with a higher prevalence in men than in women similar to that for DVT, which were 13% and 7%, respectively. Being male is an independent risk factor for PE, which has been found consistently in a number of observational studies (57). Increased hemoglobin is a known risk factor for VTE, with generally higher hemoglobin levels in men potentially being one of the reasons why men are more likely to develop VTE. In summary, VTE, encompassing DVT and PE, is a critical complication in cancer patients, especially lung cancer patients. Therefore, close clinical monitoring, accurate risk assessment, and appropriate prophylactic measures need to be taken in such patients. In addition, gender and hemoglobin level, as important risk factors, should not be ignored in clinical management.

This study has a number of limitations that need to be acknowledged. Although the qualities of our included studies were examined using the NOS scale, with an average score of 7.486 being obtained, indicating a high overall quality, the use of ICD-9 and ICD-10 codes were only used in five studies for diagnosing VTE. As most of the other 30 studies combined medical history, physical examination and imaging, the inconsistency observed in diagnosing VTE between different studies may have introduced significant bias. In addition, some of the studies dated back to the mid-2000s. As such, the observed prevalence of VTE in patients with lung cancer may not apply to patients undergoing more up-to-date contemporary individualized treatments, including targeted therapy and immunotherapy from the early 2010s onwards. Additionally, since the literature we included did not provide sufficient data to support an analysis of the incidence of VTE associated with different types of lung cancer, we did not proceed with graphical representation or further analysis of this aspect.

In conclusion, our meta-analysis demonstrates that the prevalence of lung cancer-related VTE is high and is gender- and region-specific. This study emphasizes the need to further explore the causes of regional differences and develop effective preventive strategies, provides an important basis for improving the recognition and management of VTE in lung cancer patients, and may lead to changes in clinical practice to reduce the risk of this serious complication.

## Limitations

In some of the literature we included, there may be instances where lung cancer patients have undergone surgery, radiotherapy, or chemotherapy, leading to a certain degree of heterogeneity in our analysis of the literature. Unfortunately, these studies did not provide sufficient data to allow for in-depth subgroup analysis, which is indeed regrettable. Therefore, risk factors related to VTE, such as surgery, radiotherapy, and chemotherapy, were not systematically analyzed in this study.

## Data Availability

The original contributions presented in the study are included in the article/**Supplementary Material**. Further inquiries can be directed to the corresponding authors.
