# Supplementary material for: Prevalence and clinical characteristics of venous thromboembolism in patients with lung cancer: a systematic review and meta-analysis
Source: Front Oncol. 2024 Aug 14;14:1405147. doi: 10.3389/fonc.2024.1405147 (PMC11350514; doi:10.3389/fonc.2024.1405147)
Supplement: Supplementary file 1 [file DataSheet1.doc]

**Sensitivity Analysis**


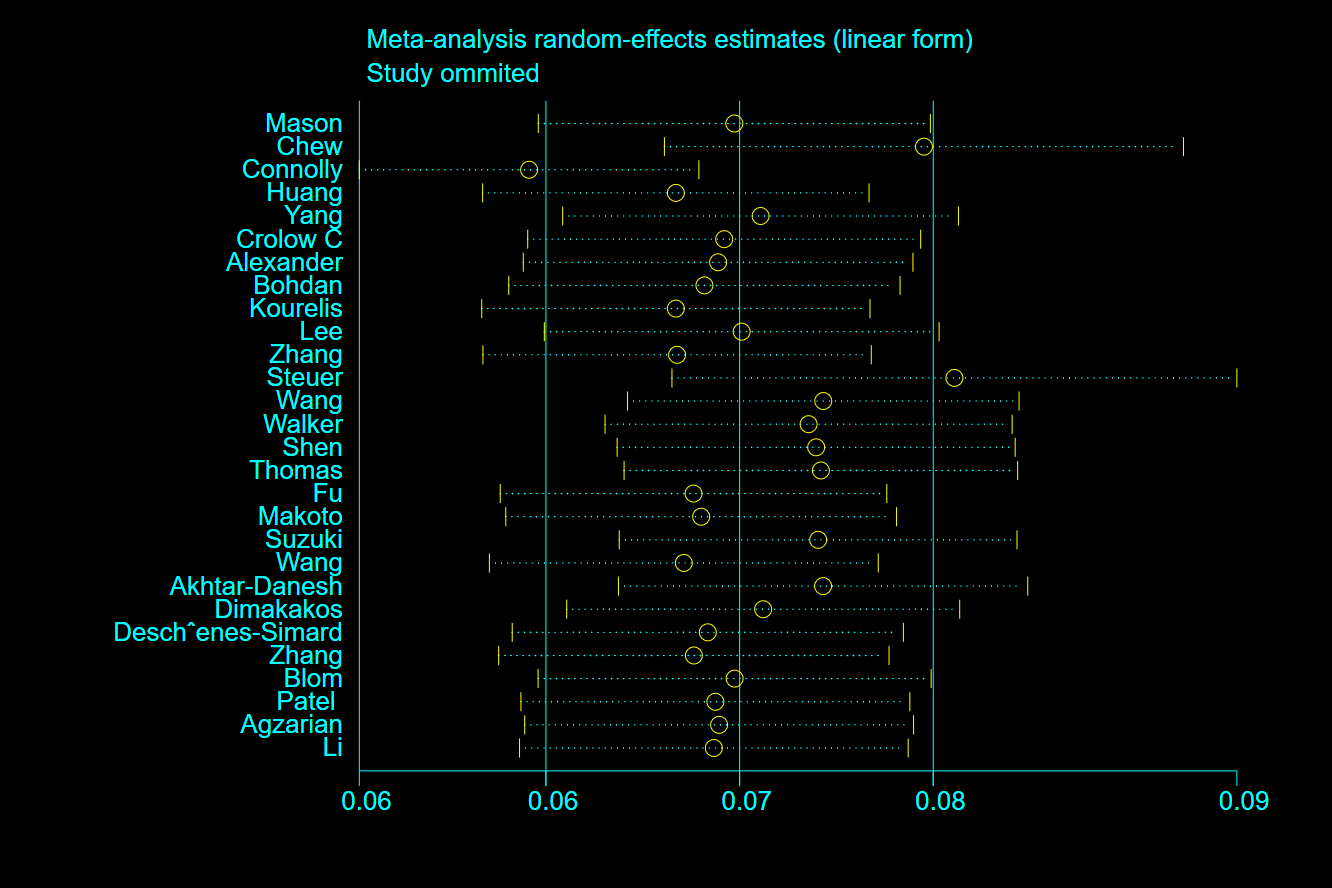


Figure 1 –Sensitivity analysis showing the prevalence of VTE in patients with lung cancer


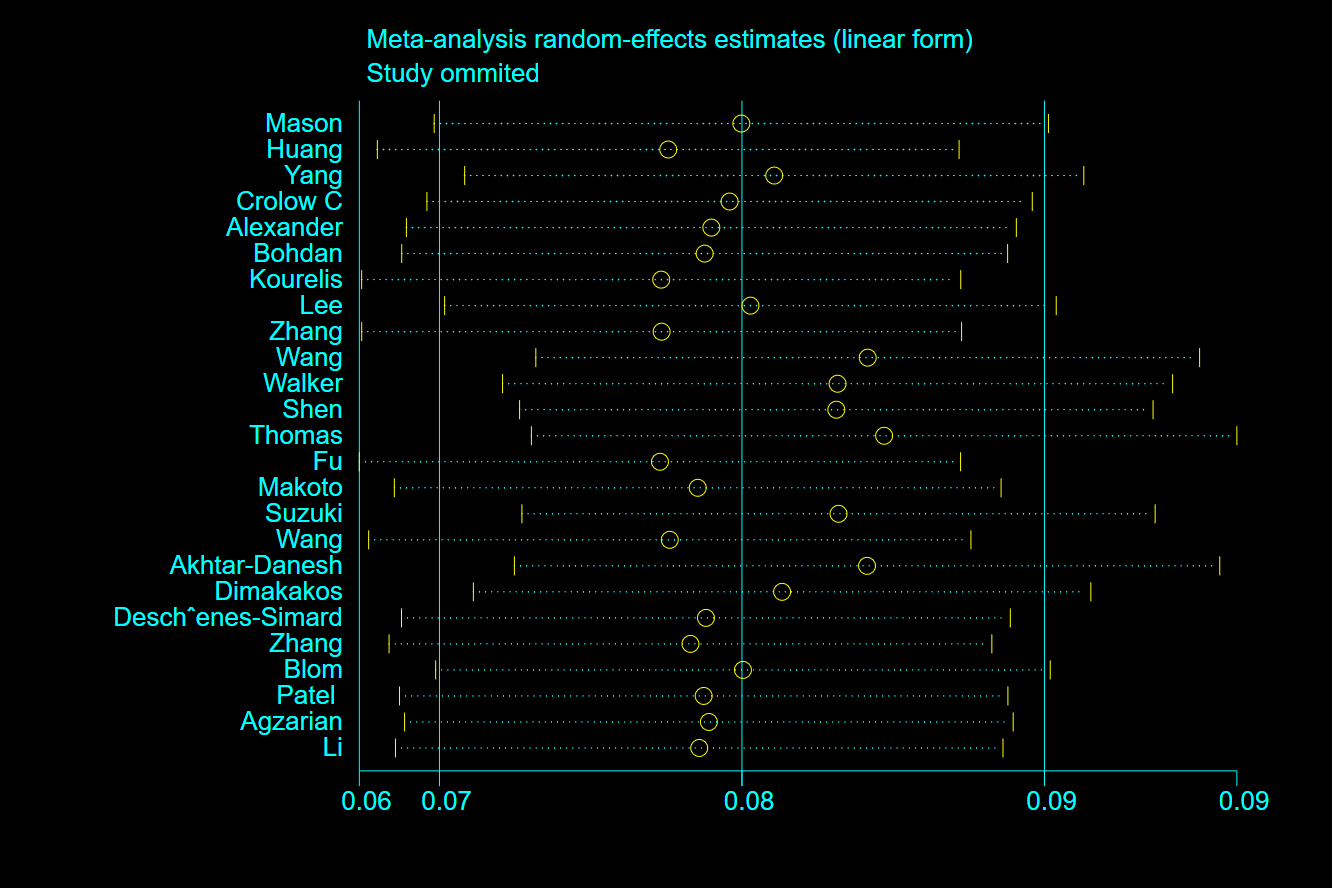


Figure 1 –Sensitivity analysis showing the prevalence of VTE in patients with lung cancer after removing three studies
