# Supplementary material for: Prevalence and clinical characteristics of venous thromboembolism in patients with lung cancer: a systematic review and meta-analysis
Source: Front Oncol. 2024 Aug 14;14:1405147. doi: 10.3389/fonc.2024.1405147 (PMC11350514; doi:10.3389/fonc.2024.1405147)
Supplement: Supplementary file 2 [file Table1.doc]

**Appendix Ⅰ**

**Details of the Literature Search Strategy**

(1) PubMed ([Jan](../../../../C:/Program%20Files%20(x86)/Youdao/Dict/8.9.6.0/resultui/html/index.html#/javascript:;) 22, 2022)

| **Search** | **Query** | **Items found** |
| --- | --- | --- |
| #1 | "Venous Thromboembolism"[MeSH Terms] | 13298 |
| #2 | "venous thromboembolism"[Title/Abstract] OR "VTE"[Title/Abstract] OR "thromboembolism"[Title/Abstract] OR "venous thrombosis"[Title/Abstract] OR "pulmonary thrombosis"[Title/Abstract] OR "pulmonary embolism"[Title/Abstract] OR "pulmonary thromboembolism"[Title/Abstract] OR "deep vein thrombosis"[Title/Abstract] | 105520 |
| #3 | #1 OR #2 | 107149 |
| #4 | "Lung Neoplasms"[MeSH Terms] | 254190 |
| #5 | "lung cancer"[Title/Abstract] OR "pulmonary cancer"[Title/Abstract] OR "lung tumor"[Title/Abstract] OR "pulmonary tumor"[Title/Abstract] OR "lung neoplasm*"[Title/Abstract] OR "lung carcinoma"[Title/Abstract] OR "pulmonary neoplasm*"[Title/Abstract] | 203238 |
| #6 | #4 OR #5 | 317592 |
| #7 | #3 AND #6 | 1463 |

(2) Embase ([Jan](../../../../C:/Program%20Files%20(x86)/Youdao/Dict/8.9.6.0/resultui/html/index.html#/javascript:;) 22, 2022)

| **Search** | **Query** | **Items found** |
| --- | --- | --- |
| #1 | 'venous thromboembolism':ti OR 'thromboembolism':ti OR vte:ti OR 'venous thrombosis':ti OR 'pulmonary thrombosis':ti OR 'pulmonary embolism':ti OR 'pulmonary thromboembolism':ti OR 'deep vein thrombosis':ti | 71084 |
| #2 | 'lung cancer':ti OR 'pulmonary cancer':ti OR 'lung tumor':ti OR 'pulmonary tumor':ti OR 'lung neoplasm*':ti OR 'lung carcinoma':ti OR 'pulmonary neoplasm*':ti | 172270 |
| #3 | #1 AND #2 | 315 |

(3) Cochrane Central Register of Controlled Trials ([Jan](../../../../C:/Program%20Files%20(x86)/Youdao/Dict/8.9.6.0/resultui/html/index.html#/javascript:;) 22, 2022)

| **Search** | **Query** | **Items found** |
| --- | --- | --- |
| #1 | MeSH descriptor: [Venous Thromboembolism] explode all trees | 748 |
| #2 | (venous thromboembolism):ti,ab,kw OR (VTE):ti,ab,kw OR (thromboembolism):ti,ab,kw OR (venous thrombosis):ti,ab,kw OR (pulmonary thrombosis):ti,ab,kw OR (pulmonary embolism):ti,ab,kw OR (pulmonary thromboembolism):ti,ab,kw OR (deep vein thrombosis):ti,ab,kw | 18630 |
| #3 | #1 OR #2 | 18630 |
| #4 | MeSH descriptor: [Lung Neoplasms] explode all trees | 8309 |
| #5 | (lung cancer):ti,ab,kw OR (pulmonary cancer):ti,ab,kw OR (lung tumor):ti,ab,kw OR (pulmonary tumor):ti,ab,kw OR (lung neoplasm*):ti,ab,kw OR (lung carcinoma):ti,ab,kw OR (pulmonary neoplasm*):ti,ab,kw | 31635 |
| #6 | #7 OR #8 | 31749 |
| #7 | #3 AND #6 | 1531 |

(4) [Web of Science](https://apps.webofknowledge.com/home.do?SID=6BQQjiiMCVa9MgFvRpC) core collection ([Jan](../../../../C:/Program%20Files%20(x86)/Youdao/Dict/8.9.6.0/resultui/html/index.html#/javascript:;) 22, 2022)

| **Search** | **Query** | **Items found** |
| --- | --- | --- |
| #1 | venous thromboembolism(Title) or VTE(Title) or thromboembolism(Title) or venous thrombosis(Title) or pulmonary thrombosis(Title) or pulmonary embolism(Title) or pulmonary thromboembolism(Title) or deep vein thrombosis(Title) | 59045 |
| #2 | lung cancer(Title) or pulmonary cancer(Title) or lung tumor(Title) or pulmonary tumor(Title) or lung neoplasm*(Title) or lung carcinoma(Title) or pulmonary neoplasm*(Title) | 185671 |
| #4 | #1 AND #2 | 778 |
